# Down-titration of biologics for the treatment of rheumatoid arthritis: a systematic literature review

**DOI:** 10.1007/s00296-017-3780-8

**Published:** 2017-08-29

**Authors:** Chak Sing Lau, Allan Gibofsky, Nemanja Damjanov, Sadiq Lula, Lisa Marshall, Heather Jones, Paul Emery

**Affiliations:** 1 Division of Rheumatology and Clinical Immunology, The University of Hong Kong, Pok Fu Lam, Hong Kong; 2 Hospital for Special Surgery and Weill Cornell Medicine, New York, NY USA; 3 Belgrade University School of Medicine, Belgrade, Serbia; 4 Envision Pharma Group, Market Access Solutions, London, UK; 5 Medical Affairs, Pfizer, Collegeville, PA USA; 6 Leeds Institute of Molecular Medicine, University of Leeds, Leeds, UK

**Keywords:** Biological therapy, Rheumatoid arthritis, Dose–response relationship, Systematic review

## Abstract

Biologic therapies have improved the management of rheumatoid arthritis (RA) and the treat-to-target approach has resulted in many patients achieving remission. In the current treatment landscape, clinicians have begun considering dose reduction/tapering for their patients. Rheumatology guidelines in Asia, Europe, and the United States include down-titration of biologics but admit that the level of evidence is moderate. We conducted a systematic literature review to assess the published studies that evaluate down-titration of biologics in RA. The published literature was searched for studies that down-titrated the following biologics: abatacept, adalimumab, certolizumab, etanercept, golimumab, infliximab, rituximab, and tocilizumab. Eligible studies included randomized controlled trials (RCTs), non-RCTs, observational, and pharmacoeconomic studies. The outcomes of interest were (1) efficacy and health-related quality of life, (2) disease flares, and (3) impact on cost. Eleven full-text publications were identified; only three were RCTs. Study results suggest that dosing down may be an option in many patients who have achieved remission or low disease activity. However, some patients are likely to experience a disease flare. Across the studies, the definition of disease flare and the down-titration criteria were inconsistent, making it difficult to conclude which patients may be appropriate and when to attempt down-titration. Studies have evaluated the practice of dosing down biologic therapy in patients with RA; however, a relatively small number of RCTs have been published. Although down-titration may be an option for some patients in LDA or remission, additional RCTs are needed to provide guidance on this practice.

**Electronic supplementary material:**

The online version of this article (doi:10.1007/s00296-017-3780-8) contains supplementary material, which is available to authorized users.

## Introduction

Rheumatoid arthritis (RA) is a chronic inflammatory rheumatic disease associated with substantial morbidity, disability, and impaired quality of life [1, 2]. The introduction of biologic therapies (referred to as biologics), including tumor necrosis factor (TNF) inhibitors, interleukin (IL)-1 and IL-6 inhibitors, and B or T cell activation inhibitors, has had a significant impact on the treatment of RA. These agents have improved the control of disease activity, enabling some patients to achieve disease remission. The current treatment strategy is “treat-to-target,” with the goal of achieving low disease activity (LDA) or remission early in the disease course [3, 4]. It is recommended that patients continue therapy to remain in LDA or remission. There are a few situations in which treatment may be stopped or suspended (drug holiday), such as surgery or pregnancy [5].

Since remission is an attainable goal, clinicians are now considering dose reduction, also referred to as dose tapering or down-titration. Recognizing this trend, the 2015 American College of Rheumatology (ACR) Guideline for the Treatment of Rheumatoid Arthritis [3] now includes downward tapering of disease-modifying antirheumatic drug (DMARD) therapy, anti-TNF therapy, non-TNF biologics, or tofacitinib in patients with established RA who have achieved remission. What is not included is a standardized definition of remission, such as how long patients should be in remission prior to tapering down the dose. This recommendation is noted to be conditional, and the level of evidence for tapering is considered moderate to very low [3].

Guidelines from the European League Against Rheumatism (EULAR) and the Asia Pacific League of Associations for Rheumatology (APLAR) also include statements that tapering of biologic therapy can be considered if a patient is in persistent or extended remission [6, 7]. The strength of these recommendations is considered moderate to high. The recommendations from these three guidelines are provided in Table 1.

**Table 1:** Comparison of three guidelines recommending dose-titration in rheumatoid arthritis

Guideline	Method for reviewing and rating quality of evidence	Recommendation	Quality of evidence
2015 ACR Guideline for the Treatment of Rheumatoid Arthritis [3]	GRADE^a^ methodology was used to evaluate the literature	For patients with established RA who are in remission:	Conditional recommendation
		Taper DMARD therapy	Low
		Taper TNF-I, non-TNF biologic or tofacitinib	Moderate to very low
EULAR recommendations for the management of RA: 2013 update [6]	Most evidence came from three SLRs; provides levels of evidence, grades of recommendations, and strengths of recommendations	If a patient persists in remission after tapering glucocorticoids, then the clinician can consider tapering bDMARDs, especially if combined with a csDMARD	LoE:^b^ 2b GoR: B SoR: 8.7 ± 1.8 Votes: 100%
APLAR RA treatment recommendations [7]	The ADAPTE framework was used to identify and review international RA guidelines, and the AGREE II instrument was used to assess the quality of the guidelines	Tapering of bDMARDs can be considered for patients in extended remission (>12 months)	LoE: 2 Strength: B

*ACR* American College of Rheumatology*, AGREE* Appraisal of Guidelines, Research and Evaluation, *bDMARD* biologic DMARD, *csDMARD* conventional synthetic DMARD, *EULAR* European League Against Rheumatism, *GoR* grade of recommendation, *GRADE* Grading of Recommendations Assessment, Development and Evaluation, *LoE* level of evidence, *RA* rheumatoid arthritis, *SLR* systematic literature review, *SoR* strength of recommendation (level of agreement), *TNF* tumor necrosis factor

^a^Group consensus was used to determine whether the recommendations were strong or conditional. A strong recommendation denotes that clinicians feel certain the benefits of an intervention are greater than the harms (or vice versa). A conditional recommendation indicates that clinicians are uncertain of the balance between benefits and harms, and/or significant variability exists in patient values and preferences. Evidence was rated as low quality or moderate to very low quality because some of the evidence was indirect (studies included discontinuation rather than tapering of therapy or patients achieved low disease activity rather than remission)

^b^LoE and GoR are based on recommendations from the Oxford Centre for Evidence-Based Medicine

Despite publication of these guidelines, information on which patients are appropriate for down-titration and the long-term consequences of such dosing (e.g., radiographic changes, subclinical inflammation or disease flares demonstrated by a return of symptoms) is limited [8]. As a result, the evidence to support down-titration may be inadequate. We conducted a systematic literature review (SLR) to evaluate the published data available on down-titration of biologics, and to assess whether sufficient evidence exists to support this treatment practice. The objectives of the SLR were to (1) compare the effect of down-titration of biologics with standard dosing of biologics on clinical efficacy and health-related quality of life (HRQoL); (2) determine how investigators defined a disease flare in different studies; (3) assess how investigators decided that certain patients were appropriate for a decreased dose of the biologic and (4) evaluate the impact of decreasing the dose on the cost of biologic therapy.

## Methods

### Search strategy

An electronic literature search was conducted, utilizing the following databases: Medline, Embase, Cochrane Central Register of Controlled Trials, Cochrane Database of Systematic Review, National Health Services Economic Evaluation Database, Health Technology Assessment Database, and Database of Abstracts of Reviews of Effects. Detailed search strategies are provided in Online Resource 1. Initially, we planned to evaluate both down-titration and up-titration of biologics in patients with RA, ankylosing spondylitis, and non-radiographic axial spondyloarthritis, and the literature search was designed in this manner. However, to provide a more focused review, this report only summarizes the literature on down-titration of biologics in patients with RA. The search was executed on February 2, 2015, and the results were limited to references published from January 2000 to February 2015 in the English language.

### Study selection

Two levels of screening were employed. At the level 1 screening, one reviewer screened the titles and abstracts of publications identified in the literature search for eligibility according to the criteria provided below. The full texts of abstracts and titles that passed level 1 screening were obtained for further review (level 2 screening). Any study ineligible for inclusion was excluded and the rationale for exclusion was documented. A second reviewer performed a quality check, and if the discrepancy level reached 5%, then the study selection was re-evaluated. Additionally, an independent reviewer screened 20% of the publications excluded at this stage and reviewed all included publications. Any discrepancies were resolved by a consensus among reviewers.

### Eligibility criteria and data extraction

Randomized controlled trials (RCTs), non-randomized controlled trials (nRCTs), observational studies, and pharmacoeconomic studies that were available as full-text publications were eligible for inclusion. RCTs were included regardless of blinding of the patients and investigators. Observational studies and nRCTs supplemented the clinical data from RCTs and captured long-term efficacy data. Pharmacoeconomic studies provided economic and outcomes data related to dose-titration. Studies were only considered if the patients were ≥18 years of age. The biologic therapies of interest included eight that were approved by regulatory authorities: abatacept (ABA), adalimumab (ADA), certolizumab pegol (CZP), etanercept (ETN), golimumab (GOL), infliximab (INF), rituximab (RTX) and tocilizumab (TCZ). Review articles, case reports, letters, and editorials were excluded, but the references were reviewed for relevant studies. Data from the included studies were collected on a customized grid to ensure that all relevant information was obtained (Online Resource 2).

### Risk of bias assessment

The quality of RCTs was assessed using the recommendations from the National Institute for Health and Clinical Excellence single technology appraisal manufacturer’s template [9]. The quality of all nRCTs and observational studies was evaluated using the Downs and Black instrument [10], and cost-effectiveness studies were evaluated using Drummond’s checklist for assessing economic evaluations [11].

## Results

### Literature search

**Fig. 1:**
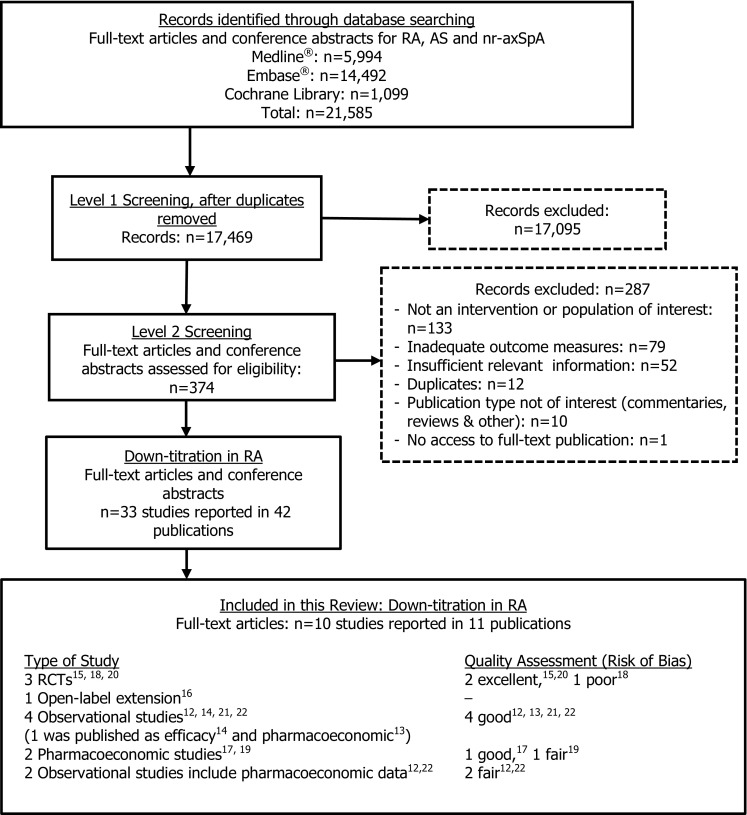
Study disposition

Following the literature search and screening process, 10 studies published in 11 full-text publications [12–22] qualified for inclusion (Fig. 1). The quality check performed by the second reviewer revealed 5 discrepancies out of 374 publications in level 2 screening, a 1% discrepancy rate that fell below the 5% level that would have prompted a re-evaluation. Qualified publications were identified for five biologics: ADA, CZP, ETN, INF, and RTX. No full-text publications evaluating down-titration in RA were identified for TCZ, ABA or GOL.

### Efficacy studies

Two RCTs and four observational studies were located for TNF inhibitors. Both RCTs evaluated ETN: PRESERVE [20] and PRIZE [15]. One RCT for RTX, the SMART study, was located [18]. In some publications, down-titration was defined as a decrease in the dose, and in others it was defined as an extension of the dosing interval. The publications in this report that evaluate efficacy are provided in Table 2.

**Table 2:** Dosing and efficacy outcomes in RA down-titration studies, full-text publications

Study citation and type	Dose of biologic	Efficacy outcome	Comments/author conclusions
Smolen et al. [20] PRESERVE RCT	OL: ETN50 + MTX qw for 36 wk, *N* = 834 then DB: ETN50 + MTX qw, *n* = 202 or ETN25 + MTX qw, *n* = 202 or PBO + MTX qw, *n* = 200	LDA at wk 88: ETN50 + MTX qw: 166/201 (82.6%) ETN25 + MTX qw: 159/201 (79.1%) PBO + MTX qw: 84/197 (42.6%) *p* < 0.0001, ETN25 or ETN50 vs PBO	Author conclusions: ETN standard or reduced dose plus MTX is more effective at maintaining LDA than MTX alone
Emery et al. [15] PRIZE RCT	OL: ETN50 + MTX qw for 52 wk, *N* = 306 then DB for 39 wk: ETN25 + MTX qw, *n* = 63 or MTX qw, *n* = 65 or PBO, *n* = 65	Sustained remission at end of DB phase: ETN25 + MTX qw: 40/63 (63%) MTX qw: 26/65 (40%) PBO: 15/65 (23%) *p* = 0.009, ETN25 + MTX vs MTX *p* < 0.001, ETN25 + MTX vs PBO	Author conclusions: Following early, aggressive treatment, some patients in remission or LDA may be considered for reduction or withdrawal of the biologic; patients should be closely monitored
Mariette et al. [18] SMART RCT	RTX 1000 mg + MTX on days 1 and 15, overall population, *N* = 224 At wk 24: RTX 1000 mg + MTX on day 1, PP *n* = 51 RTX 1000 mg + MTX on days 1 and 15, PP *n* = 49	Over 104 wks, the adjusted mean difference in DAS28-CRP AUC was 51.4 (95% CI −131.2 to 234). This was within the non-inferiority margin of 20% of the reference data (mean ± SD = 2218 ± 967; 20% = 444), indicating non-inferiority between the two doses	Author conclusions: For patients with a EULAR good or moderate response, decreasing the subsequent dose of RTX is non-inferior to administering the standard dose
Keystone et al. [16] Open-label extension of RAPID 1	400 mg CZP q2w decreased to 200 mg q2w, *N* = 436	Improvements in ACR response rates and DAS28-ESR were maintained over 192 weeks	–
Borras-Blasco [12] Observational	*N* = 24 pts with DAS28 <2.6 switched from ETN50 qw to ETN25 qw	*n* = 17 pts have continued ETN25 for a median of 3.5 ± 2.5 year *n* = 7 pts discontinued after 1.8 ± 1.2 year: *n* = 2 pts had adverse event *n* = 5 pts flared: 4 of these resumed ETN50 and one switched to ADA; all returned to clinical remission	Small number of patients in study Author conclusions: ETN25 may be considered in patients who have maintained remission on ETN50 for ≥1 year and have had slow worsening of structural changes. However, the appropriate patients have not been defined
de la Torre et al. [13] and de la Torre et al. [14] Observational	ADA Standard dose (S): *n* = 39 Reduced dose (R): *n* = 14 ETN: (S): *n* = 59 (R): *n* = 22 INF: (S): *n* = 16 (R): *n* = 2	Remission (DAS28 <2.6) ADA (S): 41.0%, ADA (R): 64.3% ETN (S): 45.8%, ETN (R): 50.0% INF (S): 37.5%, INF (R): 50.0% LDA (DAS28 2.6–3.2) ADA (S): 20.5%, ADA (R): 7.1% ETN (S): 10.2%, ETN (R): 27.3% INF (S): 6.3%, INF (R): 0% MDA (DAS28 3.2–5.1) ADA (S): 33.3%, ADA (R): 21.4% ETN (S): 37.3%, ETN (R): 9.1% INF (S): 43.8%, INF (R): 0% HDA (DAS28 >5.1) ADA (S): 5.1%, ADA (R): 7.1% ETN (S): 6.8%, ETN (R): 13.6% INF (S): 12.5%, INF (R): 50%	Efficacy was measured at 1 visit; patients had been receiving an anti-TNF for ≥12 months; dose taper was allowed for patients in remission or LDA ≥12 months Author conclusions: For patients who have responded clinically, it is reasonable to attempt a dose decrease; controlled trials are needed to determine when the dose can be titrated and which patients are appropriate
van den Bemt [21] Observational	*N* = 18 pts decreased INF from 5 mg/kg to 3 mg/kg and were followed for 3 infusions	16/18 successfully down-titrated 1/18 had persistent flare following first low-dose infusion. The flare subsided after the INF dose was increased 1/18 discontinued due to adverse event	Small number of patients in study; three infusions may not allow enough time to assess progression of RA activity Author conclusions: Most patients can decrease the dose of INF from 5 mg/kg to 3 mg/kg; the DAS28 score should be monitored
van der Maas [22] Observational	*N* = 51 pts attempted down-titration of INF. From 3 mg/kg dose decreased by 25% every 8–12 wk for 1 year until discontinued or disease flare	23/51 (45%) successfully down-titrated: 3 decreased by 25% 12 decreased by 50% 8 decreased by 75% 8/51 (16%) were able to stop INF 20/51 (39%) failed down-titration due to flare	Author conclusions: Most patients with stable LDA can decrease or discontinue INF

*ADA* adalimumab, *AUC* area under the curve, *CI* confidence interval, *CRP* C-reactive protein, *CZP* certolizumab, *DAS28* disease activity score calculated in 28 joints, *DB* double-blind, *ESR* erythrocyte sedimentation rate, *ETN* etanercept, *EULAR* European League Against Rheumatism, *HDA* high disease activity, *INF* infliximab, *LDA* low disease activity, *MDA* moderate disease activity, *MTX* methotrexate, *OL* open-label, *PBO* placebo, *PP* per protocol, *qw* once weekly, *q2w* every other week, *RCT* randomized controlled trial, *RTX* rituximab

#### TNF inhibitors

The PRESERVE trial evaluated whether patients could maintain LDA following down-titration or withdrawal of ETN [20]. Patients had moderate disease activity (DAS28 >3.2 and ≤5.1) despite methotrexate (MTX) therapy. A total of 834 patients received ETN 50 mg (ETN50) weekly (qw) + MTX qw during an initial 36-week open-label period. Patients who achieved sustained LDA (mean DAS28 ≤3.2 from weeks 12 to 36 and DAS28 ≤3.2 at week 36, *n* = 604) were randomized to receive one of three treatments (ETN50 + MTX qw, *n* = 202; ETN25 + MTX qw, *n* = 202; or placebo (PBO) + MTX qw, *n* = 200) for a double-blind period of 52 weeks. The primary endpoint was the proportion of patients maintaining LDA in the ETN50 + MTX and PBO + MTX groups at week 88. The proportion of patients maintaining LDA at week 88 in the ETN25 + MTX group was a conditional primary endpoint.

Significantly more patients in the ETN50 + MTX group achieved the primary endpoint than in the PBO + MTX group: 166/201 (82.6%) vs 84/197 (42.6%); *p* < 0.0001 (Table 2) [20]. The proportion of patients maintaining LDA in the ETN25 + MTX group was 159/201 (79.1%); *p* < 0.0001 vs PBO. The efficacy of the ETN50 and ETN25 doses was not directly compared. Health Assessment Questionnaire (HAQ) scores were significantly lower in the ETN50 and ETN25 groups compared with PBO, with HAQ mean [standard deviation (SD)] scores of 0.5 (0.5) for ETN50, 0.6 (0.5) for ETN25, and 0.8 (0.6) for PBO; *p* < 0.0001 for both ETN doses vs PBO.

The number of patients who discontinued during the double-blind period due to unsatisfactory responses was 4, 11, and 43 in the ETN50, ETN25, and PBO groups, respectively. The authors concluded that both standard and reduced doses of ETN in combination with MTX are more effective in maintaining LDA than MTX alone following discontinuation of ETN.

The PRIZE study enrolled patients with early active disease who had not been previously treated with MTX or biologic therapy [15]. All patients received open-label ETN50 + MTX qw for 52 weeks. Then, patients who qualified at weeks 39 and 52 received double-blind ETN25 + MTX, or MTX only, or PBO. The qualification criteria were DAS28 ≤3.2 at week 39 and DAS28 <2.6 at week 52. After 39 weeks of double-blind therapy, patients with DAS28 ≤3.2 were withdrawn from treatment. The primary endpoint was sustained remission (DAS28 <2.6) at weeks 24 and 39 of the double-blind phase.

The primary endpoint was achieved by significantly more patients in the ETN25 + MTX group [40/63, (63%)] than in the MTX only [26/65 (40%); *p* = 0.009] or PBO group [15/65 (23%); *p* < 0.001] (Table 2) [15]. At the end of the double-blind phase, the percentage of patients with a normal HAQ score was 78, 72, and 45% for the ETN25 + MTX, MTX only, and PBO groups, respectively; *p* < 0.001 for ETN25 + MTX vs PBO. The authors concluded that after early, aggressive treatment to achieve remission or LDA, a reduction in dose or withdrawal of the biologic may be acceptable in some patients, especially those with sustained ACR-EULAR Boolean-based remission.

In the open-label extension of the RAPID 1 study, patients initially received CZP 400 mg (CZP400) every other week (q2w) + MTX [16], then the dosing was changed to CZP200 q2w. Improvements in DAS28-ESR and ACR response rates were maintained over 192 weeks following the dose decrease (Table 2).

Four observational studies evaluated dosing down a TNF inhibitor in patients with RA (Table 2). A retrospective observational study by Borrás-Blasco et al. [12] evaluated patients who had achieved and maintained DAS28 <2.6 for at least 1 year on ETN50 qw and then were switched to ETN25 qw. At study end, 17 of 24 patients were continuing ETN25; the median treatment duration was 3.5 ± 2.5 years. Seven patients had discontinued ETN25, five of them due to disease flare, defined as DAS28 >2.6 (Table 2). Four of the patients with flare resumed ETN50, and one switched to ADA; all were able to return to clinical remission.

A cross-sectional study by de la Torre et al. [13, 14] evaluated patients who had been receiving a TNF inhibitor for ≥12 months. Down-titration of the dose was allowed for patients who had achieved remission or LDA (DAS28 <2.6 or <3.2, respectively) for ≥12 months. Efficacy was evaluated at one visit for a total of 195 patients who were receiving a standard, reduced, or escalated dose of ADA (*n* = 39, 14, 3, respectively), ETN (*n* = 59, 22, 0), or INF (*n* = 16, 2, 40), Table 2. Based on a comparison of the response rates at standard and reduced doses, the authors concluded that it is acceptable to attempt to decrease the dose once patients have achieved a clinical response.

A prospective observational study by van den Bemt et al. [21] evaluated patients who were receiving INF 5 mg/kg and had stable disease activity and DAS28 ≤5.1. The 18 patients had been receiving INF 5 mg/kg for a mean (SD) of 19 (14) months every 6.1 (1.5) weeks. The dose was decreased to 3 mg/kg with the dosing interval left unchanged. Patients were followed for three infusions; disease flare was defined using reversed EULAR response criteria (an increase in DAS28 >1.2 or an increase in DAS28 >0.6 and a current DAS28 >5.1). A total of 16/18 patients were successfully down-titrated (Table 2).

In another prospective observational study, van der Maas et al. [22] included patients receiving INF 3 mg/kg with stable LDA (DAS28 <3.2) and stable therapy for ≥6 months. The dose of INF was decreased by 25% every 8–12 weeks until it was discontinued or the patient experienced a disease flare. Flare was defined as an increase in DAS28 ≥1.2 from baseline on two subsequent visits with ≥2 weeks between visits; after the patient reached DAS28 >3.2, then flare was defined as an increase in DAS28 ≥0.6. Of the 51 patients who were included, 23 (45%) were successfully down-titrated, 8 (16%) were able to stop INF, and 20 (39%) failed down-titration due to flare (Table 2). The difference in HRQoL before and after down-titration, as measured by the EuroQoL 5-dimensions (EQ5D), was not statistically significant.

#### Rituximab

The SMART study was an open-label non-inferiority study in patients with an inadequate response to TNF inhibitors [18]. All patients received RTX 1000 mg + MTX for 2 doses, *N* = 224. At week 24, patients with a moderate or good EULAR response were randomized to receive RTX 1000 mg for 1 or 2 doses (Table 2). The primary endpoint was DAS28-CRP area under the curve (AUC) over 104 weeks; the non-inferiority margin was defined as 20% of the DAS28-CRP AUC of the reference data (mean = 2218; 20% = 444). Over 104 weeks, the adjusted mean difference in DAS28-CRP AUC was 51.4 (95% CI −131.2–234), indicating non-inferiority between the two doses.

### Economic outcomes

Economic data were reported for ADA, ETN, INF, and RTX, with most studies reporting a decrease in cost with dosing down. Kobelt [17] conducted a cost-effectiveness analysis based on the dosing in the PRESERVE trial. An economic model compared the costs of standard and reduced-dose ETN to MTX monotherapy in Sweden. In the model, patients have LDA at the start. Data were extrapolated out 10 years using information from the Swedish RA registry. The authors estimated the cost per quality-adjusted life-year (QALY) gained with ETN25 vs MTX ranged from €14,000 to €29,000, depending on how long ETN was administered. ETN25 had slightly lower costs and similar effectiveness compared to ETN50.

In a US retrospective cost analysis, Nair et al. [19] utilized the MarketScan database to compare the medical and pharmacy costs of INF resulting from dose decrease, dose increase, or no dose change in the first year of treatment. The authors found that 29.9 and 24.7% of the 1678 patients with commercial insurance maintained and decreased their dose, respectively, compared with 17.5 and 43.2% of the 616 patients with government insurance. Patients with commercial insurance who maintained their dose had significantly lower medical costs (per member per year) than those who decreased their dose ($21,011 vs $27,697, respectively; *p* = 0.01) and significantly lower pharmacy costs ($2072 vs $3229, *p* = 0.01). For patients with government insurance, costs were also lower for maintaining vs decreasing the dose (medical: $15,967 vs $18,446; respectively, *p* = ns; pharmacy: $2380 vs $3477, *p* = 0.01). Patients with a decreased vs maintenance dose had a significantly higher number of inpatient admissions, physician visits, laboratory/diagnostic tests, and prescriptions.

In contrast, three observational studies (Table 2) found that costs decreased with a lower dose of biologic therapy. Van der Maas et al. [22] found that down-titration and discontinuation of ETN resulted in a mean decrease in cost per patient over 1 year of €3474 (95% CI €2457–€4492). If these results were extended to additional years, the authors estimated an annual cost savings of €5689 per patient. Borras-Blasco et al. [12] calculated the direct cost savings from using ETN25 rather than ETN50 to be €404,008 during the study period of January 2006 to June 2013. Finally, in the study by de la Torre et al. [13], the low mean doses of ETN (44.9 mg qw) and ADA (37.4 mg q2w) resulted in a savings per patient/year of €1223.6 (−10.3%) for ETN and €839.7 (−6.5%) for ADA.

## Discussion

The current treatment strategy in RA is “treat-to-target” early in the disease course to prevent permanent joint damage [3, 4, 23–25]. Increasingly, clinicians are considering down-titration, and this consideration is largely influenced by cost reduction [8]. This review assessed the biologic dose-titration literature in RA to determine whether sufficient evidence exists to support the practice of down-titration. Clinical studies evaluating dosing down of biologics are beginning to be published, and the authors are reporting that decreasing the dose of the biologic in patients in remission or LDA has some success. However, the study results demonstrate that some patients are unable to remain in remission and will experience a disease flare.

Sustained remission is the treatment goal in RA to prevent permanent joint damage [4]. Although some patients may tolerate a reduction in the biologic dose, in other patients, tapering biologic therapy after achieving remission may increase the risk of disease flare and impact radiographic progression. RCT data are limited to date, and additional studies are needed to support and guide the practice of dosing down biologic therapy. Finally, although a decreased biologic dose may lower drug costs, patients in sustained remission will likely have lower healthcare utilization costs than patients with active disease. Since we cannot predict which patients will tolerate down-titration and which ones will experience a disease flare, this practice may not be in the best interest of either the healthcare system or the patient [13].

This review of the literature identified several limitations in the available evidence and discrepancies between the publications. Few full-text publications were located, and many of these studies were observational rather than RCTs. The published studies were variable in design and included a wide range of baseline disease activity and durations, and prior DMARD and biologic use [8, 26]. A standardized definition of treatment success or failure was lacking, and few studies used established definitions of disease flare. In many studies, there was no statistical comparison between the standard and decreased dose; the only comparison was with PBO or MTX. The quality or risk of bias for most of the studies was graded as good to excellent (7 of 11); however, 3 studies were considered fair and 1 was considered to be of poor quality. As a result, it was difficult to draw a conclusion as to when and in which patients it may be appropriate to attempt down-titration.

Based on the literature review, we have developed several recommendations for investigators who are designing a dose-titration study (Table 3).

**Table 3:** Recommended elements to include when designing a dose-titration study

1. A homogeneous patient population with similar levels of disease activity, duration of disease, and prior use of DMARDs and biologics
2. An established definition of disease flare
3. A clear statement on how improvement or relapse is being measured
4. Established definitions of low disease activity, remission, moderate disease activity, partial remission, high disease activity, and/or relapse
5. A statistical comparison of the efficacy of the standard dose and the titrated dose
6. Safety and pharmacoeconomic data

## Conclusion

Clinical studies have shown that down-titration of biologic therapy in patients with RA who have achieved remission or LDA is successful in many patients. Additionally, most studies that conducted a cost analysis determined that costs decrease when the dose of the biologic decreases. However, the study results also demonstrated that following down-titration, some patients are unable to maintain remission or LDA and experience a disease flare. Since their RA is no longer under control, these patients may be at risk for joint damage. Additional well-designed, high-quality RCTs that use standardized definitions of remission and disease flare are needed to provide guidance to clinicians on whether dosing down biologic therapy is appropriate for particular patients.

## Electronic supplementary material

Below is the link to the electronic supplementary material.

Supplementary material 1 (PDF 177 kb)
