# Supplementary material for: Down-titration of biologics for the treatment of rheumatoid arthritis: a systematic literature review
Source: Rheumatol Int. 2017 Aug 29;37(11):1789–98. doi: 10.1007/s00296-017-3780-8 (PMC5645436; doi:10.1007/s00296-017-3780-8)
Supplement: Supplementary file 1 — Supplementary material 1 (PDF 177 kb) [file 296_2017_3780_MOESM1_ESM.pdf]

Online Resources

Down-titration of biologics for the treatment of rheumatoid arthritis: A systematic literature review

Rheumatology International

Chak Sing Lau; Allan Gibofsky; Nemanja Damjanov; Sadiq Lula; Lisa Marshall; Heather Jones; Paul Emery

Corresponding Author:
Chak Sing Lau
Division of Rheumatology and Clinical Immunology
The University of Hong Kong
Hong Kong
Email: CSLau@hku.hk

Online Resource 1.

Electronic database search strategy: Ovid MEDLINE In-Process and other non-indexed citations and Ovid MEDLINE 1946 to present

Search number	Search terms	Parameter	Hits
1	exp rheumatoid arthritis/ or exp Spondylitis, Ankylosing/	Disease	105,527
2	rheumatoid arthritis.tw.		81,923
3	((rheumatoid or reumatoid or revmatoid or rheumatic or rheumatic or revmatic or rheumat$ or reumat$ or revmarthrit$) adj3 (arthrit$ or artrit$ or diseas$ or condition$ or nodule$)).tw.		101,137
4	(felty$ adj2 syndrome).tw.		693
5	(caplan$ adj2 syndrome).tw.		116
6	(sjogren$ adj2 syndrome).tw.		11,842
7	(sicca adj2 syndrome).tw.		773
8	still$ disease.tw.		1,709
9	(ankylosing spondylitis or 'AS').tw.		9,955
10	(bekhterev or bechterew or marie strumpell or rheumatoid spondylitis or ankylos* or spondyl* or axial).tw.		93,440
11	(Non$radiographic or radiograph* or nr-axSpA).tw.		163,447
12	or/1-11		381,853
13	(Abatacept or orencia or BMS-224818 or BMS 188667).mp.	Intervention	2,749
14	(Adalimumab or Humira or Hum?ra).mp.		4,501
15	(certolizumab or CDP870 or CDP 870 or PHA738144 or PHA 738144 or Cimzia).mp.		598
16	(certolizumab adj2 pegol).mp.		471
17	(Etanercept or Enbrel or or ETA or ETN).mp.		17,263
18	(golimumab or CNTO 148 or CNTO148 or Simponi).mp.		406
19	(Infliximab or Remicade or Avakine).mp.		9,843
20	(Rituximab or rituxan or Mabthera).mp.		13,601
21	(Tocilizumab or actemra or atlizumab).mp.		1,115
22	or/13-21		43,618
23	12 and 22		7,887
24	randomized controlled trial.pt.		401,556
25	controlled clinical trial.pt.		90,822
26	randomi?ed.ab.		385,775
27	placebo.tw.		169,359
28	drug therapy.fs.		1789,870
29	clinical trials as topic.sh.		176,781
30	randomly.ab.		230,591
31	trial.ab.		336,214

32	groups.ab.	Study design	1448,661
33	(crossover or cross-over or cross over).tw.		64,276
34	((singl$ or double$ or triple$ or treble$) and (blind$ or mask$)).tw,sh.		159,960
35	(case control or case-control).ti,ab.		85,986
36	((follow up or follow-up) adj (study or studies)).ti,ab.		39,602
37	(Longitudinal or retrospective or prospective or comparative or cohort or cross sectional or cross-sectional).ti,ab.		1351,520
38	((observ$ or registry) adj3 (study or studies)).ti,ab.		106,302
39	or/24-38		4,672,267
40	(exp Costs/ and cost analysis/) or cost-benefit analysis/		104,776
41	Economics, Pharmaceutical/ or Economics, Medical/ or Economics/ or exp Economics, Hospital/ or Economics, Dental/ or Economics, Nursing/		64,363
42	(economic$ or cost or costs or costly or costing or price or prices or pricing or pharmacoeconomic$).ti,ab.		502,180
43	(expenditure$ not energy).ti,ab.		20,171
44	(value adj1 money).ti,ab.		30
45	budget$.ti,ab.		20,166
46	or/40-45		594,612
47	((energy or oxygen) adj cost).ti,ab.		3,070
48	(metabolic adj cost).ti,ab.		914
49	((energy or oxygen) adj expenditure).ti,ab.		18,721
50	or/47-49		21,915
51	46 not 50		589,758
52	Quality-Adjusted Life Years/		7,642
53	exp "Outcome and Process Assessment (Health Care)"/ or exp "Outcome Assessment (Health Care)"/ or exp Treatment Outcome/ or exp "Quality of Life"/		869,944
54	((sf or short form or shortform) and "6").tw.		8,476
55	(EuroQol or standard gamble or time trade off or time tradeoff or TTO or EQ5D or EQ-5D or health utilit$ index).tw.		6,594
56	((Health and utilit* and index) or HUI* or SF-6D or sf6* or sf 6* or short form 6* or shortform 6* or sf six or sfsix or short form six or shortform six or QALY or quality adjusted life year* or quality-adjusted life year or quality adjusted life-year* or quality-adjusted life-year* or SF-36 or sf36 or sf 36 or short form 36 or shortform 36 or sf thirtysix or sf thirty six or shortform thirtysix or shortform thirty six or short form thirtysix or short form thirty-six or short form thirty six or short form health survey or willingness to pay).tw.		34,254
57	((utilit* and score*) or (utilit* and weight*) or Rosser or (health and utilit*) or (utilit* and value) or disutility*).tw.		36,250
58	(outcome and measure$).tw.		245,112
59	(health and outcome$).tw.		166,310
60	or/52-59		1,175,590

61	39 or 51 or 60		5,556,250
62	23 and 61		6,810
63	(animals not (humans and animals)).sh.		4,004,891
64	61 not 63		4,927,391
65	23 and 64		6,762
66	limit 65 to English language		6,051
**67**	**limit 66 to yr="2000 -Current"**		**5,994**

Electronic database search strategy: Embase 1988 to present

Search number	Search terms	Parameter	Hits
1	exp rheumatoid arthritis/or exp spondylarthritis/ or exp ankylosing spondylitis/ or exp spondyloarthropathy/	Disease	128,537
2	((rheumatoid or reumatoid or revmatoid or rheumatic or reumatic or revmatic or rheumat$ or reumat$ or revmarthrit$) adj3 (arthrit$ or artrit$ or diseas$ or condition$ or nodule$)).tw.		103,828
3	(felty$ adj2 syndrome).tw.		386
4	(caplan$ adj2 syndrome).tw.		42
5	(sjogren$ adj2 syndrome).tw		12,004
6	(sicca adj2 syndrome).tw.		734
7	still$ disease.tw		1,811
8	(ankylosing spondylitis or 'AS').tw.		12,448
9	(bekhterev or bechterew or marie strumpell or rheumatoid spondylitis or ankylos* or spondyl* or axial).tw.		91,229
10	(Non$radiographic or radiograph* or nr-axSpA).tw.		156,014
11	or/1-10		380,303
12	exp abatacept/ or exp adalimumab/ or exp certolizumab pegol/ or exp etanercept/ or exp golimumab/ or exp infliximab/ or exp rituximab/ or exp tocilizumab/ or exp ustekinumab/	Intervention	83,658
13	(Abatacept or orencia or or BMS-224818 or BMS 188667).mp.		5,473
14	(Adalimumab or Humira or Hum?ra).mp.		17,099
15	(certolizumab or CDP870 or CDP 870 or PHA738144 or PHA 738144 or Cimzia).mp.		3,384
16	(certolizumab adj2 pegol).mp.		3,049
17	(Etanercept or Enbrel or ETA or ETN).mp.		29,146
18	(golimumab or CNTO 148 or CNTO148 or Simponi).mp.		2,495
19	(Infliximab or Remicade or Avakine).mp.		31,318
20	(Rituximab or rituxan or Mabthera).mp.		43,093
21	(Tocilizumab or actemra or atlizumab).mp.		4,406
22	or/12-21		95,308

23	11 and 22		24,031
24	(random$ or placebo$).ti,ab		946,307
25	clinical trial/	Study design	791,848
26	random$.tw.		879,921
27	randomized controlled trial/		340,282
28	trial$.tw.		789,789
29	controlled study/		4,407,623
30	double blind procedure/		109,470
31	placebo$.tw.		188,209
32	(singl$ adj (blind$ or mask$)).tw.		14,128
33	(crossover$ or cross-over$).ti,ab.		63,406
34	(double$ adj (blind$ or mask$)).tw.		130,046
35	Crossover Procedure/		40,750
36	Single Blind Procedure/		19,125
37	((triple$ or treble$) adj (blind$ or mask$)).tw.		420
38	(Longitudinal or retrospective or prospective or comparative or cohort or cross sectional or cross-sectional).ti,ab.		1,589,551
39	((observ$ or registry) adj3 (study or studies)).ti,ab.		137,053
40	(case control or case-control).ti,ab.		94,143
41	or/24-40		6,547,318
42	health-economics/ or exp economic-evaluation/ or exp health-care-cost/ or exp pharmacoeconomics/		447,193
43	(econom$ or cost or costs or costly or costing or price or prices or pricing or pharmacoeconomic$).ti,ab.		570,579
44	(expenditure$ not energy).ti,ab.		21,961
45	(value adj2 money).ti,ab.		1,398
46	budget$.ti,ab.		21,828
47	or/42-46		835,491
48	(metabolic adj cost).ti,ab.		812
49	((energy or oxygen) adj cost).ti,ab.		2629
50	((energy or oxygen) adj expenditure).ti,ab.		19,318
51	or/48-50		22,045
52	47 not 51		831,062
53	exp quality adjusted life year/		13,001
54	exp treatment outcome/		1,010,827
55	exp "quality of life"/		281,076
56	exp outcome assessment/		258,518
57	((sf or short form or shortform) and "6").tw.		12,180
58	(EuroQol or standard gamble or time trade off or time tradeoff or TTO or		9,946

	EQ5D or EQ-5D or health utilit$ index).tw.		
59	((Health and utilit* and index) or HUI* or SF-6D or sf6* or sf 6* or short form 6* or shortform 6* or sf six or sfsix or short form six or shortform six or QALY or quality adjusted life year* or quality-adjusted life year or quality adjusted life-year* or quality-adjusted life-year* or SF-36 or sf36 or sf 36 or short form 36 or shortform 36 or sf thirtysix or sf thirty six or shortform thirtysix or shortform thirty six or short form thirtysix or short form thirty-six or short form thirty six or short form health survey or willingness to pay).tw.		45,531
60	((utilit* and score*) or (utilit* and weight*) or Rosser or (health and utilit*) or (utilit* and value) or disutility*).tw.		47,794
61	or/53-60		1,285,715
62	41 or 52 or 61		7,680,496
63	23 and 62		15,659
64	(animal$ not human$).sh,hw.		2,575,972
65	62 not 64		6,222,937
66	23 and 65		15,542
**67**	Limit 46 to (English language and yr="2000-Current")		**14,492**

Electronic database search strategy: Cochrane Library

Search number	Search terms	Parameter	Hits
1	MeSH descriptor: [Arthritis, Rheumatoid] explode all trees	Disease	4,149
2	MeSH descriptor: [Spondylitis, Ankylosing] explode all trees		442
3	rheumatoid arthritis:ti,ab,kw		6,664
4	((rheumatoid or 6eneraliza or revmatoid or rheumatic or 6eneraliz or revmatic or rheumat* or reumat* or revmarthrit*) near/3 (arthrit* or artrit* or diseas* or condition* or nodule*)):ti,ab,kw		7,543
5	(felty* near/2 syndrome).ti,ab,kw		1
6	(caplan* near/2 syndrome). Ti,ab,kw		1
7	(sjogren* near/2 syndrome). Ti,ab,kw		3
8	(sicca near/2 syndrome). Ti,ab,kw		1
9	still* disease. Ti,ab,kw		23
10	ankylosing spondylitis:ti,ab,kw		734
11	(bekhterev or bechterew or marie strumpell or rheumatoid spondylitis or 6enerali* or spondyl* or axial).ti,ab,kw		27
12	(Non$radiographic or radiograph* or nr-axSpA).ti,ab,kw		113
13	#1 or #2 or #3 or #4 or #5 or #6 or #7 or #8 or #9 or #10 or #11 or #12		8,425
14	(Abatacept or orencia or BMS-224818 or BMS 188667):ti,ab,kw		183
15	(adalimumab or humira or hum?ra):ti,ab,kw		526
16	(certolizumab or CDP870 or CDP 870 or PHA738144 or PHA 738144		88

	or Cimzia):ti,ab,kw	Intervention	
17	(certolizumab near/2 pegol):ti,ab,kw		78
18	(Etanercept or Enbrel or ETA or ETN):ti,ab,kw		909
19	(golimumab or CNTO 148 or CNTO148 or Simponi):ti,ab,kw		114
20	(Infliximab or Remicade or Avakine): ti,ab,kw		827
21	(Tocilizumab or actemra or atlizumab):ti,ab,kw		120
22	(Rituximab or rituxan or Mabthera):ti,ab,kw		1,005
23	#14 or #15 or #16 or #17 or #18 or #19 or #20 or #21 or #22		3,238
24	#13 and #23		1,113
**25**	#24 publication year from 2000, in Cochrane Reviews (reviews and protocols), other reviews, trials, technology assessments and economic evaluations		**1,099**

Online Resource 2. Data Extraction Grid

**Study information**	**Information extracted**
Study characteristics	Author<br>Study name<br>Disease<br>Intervention<br>Study design<br>Study objective<br>Trial length<br>Study duration<br>Study size<br>Study setting<br>Detail of cross-over (including timeline of cross-over)<br>Number of patients (per treatment arm)<br>Study limitations<br>Authors' conclusions and comments
Treatment data	Dose<br>Frequency<br>Concomitant medication<br>Route of administration
Baseline characteristics	Intervention and posology<br>Sample size<br>Study duration<br>Age<br>Gender<br>Disease activity and duration<br>Number of previous DMARDs by dose and frequency of administration<br>Biologic-naive patients<br>Baseline disease characteristics such as DAS28, CRP, ESR, HAQ, ASDAS, BASDAI, mTSS, etc.
Efficacy and radiographic progression outcomes	Tender joint count<br>Swollen joint count<br>Physician global assessment of disease using 10-cm visual analog scale or Likert scale<br>Acute phase reactants such as ESR or CRP

	Radiographic progression as measured by Sharp scores, mTSS, or GmSS<br>ACR 20/50/70<br>EULAR response<br>CDAI response<br>SDAI<br>ASDAS response<br>ASAS response<br>BASDAI
Health-related quality of life (HRQoL) and economic outcomes	Patient's assessment of functional ability as measured by a validated scale such as the HAQ, ADL, or SF-36 (physical) (HRQoL outcomes)<br>Indirect and direct costs (economic outcomes)
